# Desiccation tolerance as a driver of environmental persistence and transmission in nosocomial bacterial pathogens

**DOI:** 10.1128/iai.00557-25

**Published:** 2026-08-04

**Authors:** Michael J. Bucher, Alejandro Rivera-Madera, Erin R. Green

**Affiliations:** 1 Department of Microbiology, The University of Chicago 456539, Chicago, Illinois, USA; 2 Committee on Microbiology, The University of Chicago 2462, Chicago, Illinois, USA; University of California at Santa Cruz, Santa Cruz, California, USA

**Keywords:** hospital-acquired infections, desiccation, transmission, desiccation tolerance

## Abstract

Hospital-acquired infections (HAIs) represent a major global health burden, leading to increased patient morbidity, mortality, and healthcare costs. These infections frequently occur following patient contact with microbial pathogens persisting on indwelling devices and other abiotic surfaces in the hospital environment. A key driver of this persistence is the ability to survive the total loss of water, a phenomenon known as desiccation tolerance. While desiccation tolerance has been studied across various kingdoms of life, comparatively little is known about the molecular mechanisms that enable bacterial pathogens to tolerate extreme water loss. In this review, we will examine the consequences of desiccation stress in bacteria and highlight conserved mechanisms by which bacterial cells tolerate drying, including spore formation, capsule synthesis, osmolyte accumulation, expression of intrinsically disordered proteins, and other protective mechanisms. We will also examine the mechanisms and consequences of desiccation tolerance in several clinically significant nosocomial pathogens and review emerging evidence that desiccation stress responses may influence other bacterial traits, such as antimicrobial resistance, polymicrobial community interactions, and virulence. Understanding the mechanisms by which nosocomial pathogens tolerate desiccation may reveal novel strategies to disrupt pathogen reservoirs and reduce future HAI transmission.

## INTRODUCTION

Hospital-acquired infections (HAIs) affect 1 in 10 patients and up to 30% of patients in intensive care units, representing a major health burden (1). A substantial proportion of these infections likely arises from transmission via contaminated surfaces, medical devices, and other components of the hospital-built environment. Consistent with this, pathogens that are commonly associated with HAIs are also frequently isolated from these abiotic surfaces (2, 3). Successful transmission, therefore, requires these pathogens to survive on hospital surfaces for extended periods. Several clinically important nosocomial pathogens, including *Acinetobacter baumannii*, *Enterococcus* spp., *Clostridioides difficile*, *Pseudomonas aeruginosa*, and *Staphylococcus aureus*, have evolved strategies to tolerate the multitude of stressors encountered in the hospital environment, which may contribute to their heightened fitness and efficient transmission (4, 5). A key example of this evolution is the increased tolerance of nosocomial pathogens to hospital disinfectants, including ethanol-based, oxidant-based, and quaternary ammonium-based biocides (6–12). Additionally, a defining feature associated with survival on abiotic surfaces is exposure to prolonged drying, which imposes significant stress on bacterial cells. The capacity of an organism to endure such conditions and return to normal function after rehydration is known as desiccation tolerance, and although it is rare, it has been observed across the tree of life, including in bacteria. In bacterial pathogens, it is hypothesized that extreme desiccation tolerance is an important driver of transmission in healthcare settings (13, 14).

Water is essential for life at all scales, comprising up to 80% of intracellular volume (15); therefore, the complete removal of water can significantly affect cell viability. Many macromolecules, including proteins, lipids, and nucleic acids, must interact with water to maintain their stable structure and function (15–19). Because of this, desiccation stress can damage any cell component that is comprised of or contains these macromolecules, making tolerance to desiccation a substantial challenge. Since desiccation induces a diversity of stressors, the resulting stress responses may broadly alter bacterial physiology, influencing other pathogenicity traits. Emerging evidence suggests that, beyond facilitating transmission, desiccation stress responses also enhance tolerance to other environmental stressors, shape host-pathogen interactions, and accelerate evolution in nosocomial pathogens. For example, the response to desiccation also increases bacterial tolerance to disinfectants (7), further preventing their removal from the hospital environment and aiding in their transmission. Similarly, it has been shown that the protective response to desiccation can alter the emergence of antibiotic resistance and influence polymicrobial community structure and virulence (7, 20–23).

Desiccation survival mechanisms are well characterized in eukaryotic organisms, such as plants, tardigrades, nematodes, and fungi (24). By contrast, the mechanisms that bacteria employ to survive desiccation remain poorly understood. Therefore, further investigation of the molecular mechanisms driving desiccation tolerance in nosocomial pathogens could inform novel approaches to mitigate their transmission and virulence. Here, we summarize the specific forms of damage that bacterial pathogens undergo during desiccation stress, highlight the general and species-specific mechanisms of desiccation tolerance observed in bacteria to date, and outline the major questions remaining in the field. It is our hope that a thorough synthesis of the existing knowledge surrounding desiccation tolerance in nosocomial pathogens will highlight these processes as promising targets for translational strategies to limit transmission in hospital environments.

## DESICCATION-ASSOCIATED DAMAGE

Because water is essential as a cofactor and the most significant molecule in biological and chemical reactions, its loss induces a wide range of stressors. During drying, organisms can simultaneously face osmotic stress, membrane destabilization, intracellular crowding, protein misfolding, DNA damage, and disruption of metabolic pathways (25). The extent of desiccation-associated damage is central to understanding why desiccation tolerance requires such a wide array of protective responses and may explain the relative rarity of desiccation-tolerant species.

In the initial stages of drying, extracellular water content decreases rapidly, which leads to an increase in the relative concentrations of extracellular solutes, causing a significant increase in osmotic pressure. To maintain the osmotic pressure gradient, water rapidly flows out of the cell, thereby inducing a wide range of cellular stressors. As water loss occurs, cell membranes shrink as intracellular volume decreases. This rapid membrane shrinkage increases permeability or leakage, thereby accelerating water flow out of the cell (26). This reduces the time that cells have to adapt to drying and can induce irreversible damage. Furthermore, bacterial cell membranes are primarily composed of phospholipids, which interact strongly with water under normal conditions (18). As water is removed from the system, the hydration layer of these phospholipids is stripped away, increasing the electrostatic repulsion between polar head groups (25, 27). This results in decreased lipid mobility, increased membrane rigidity, and a significant increase in the membrane phase transition temperature (*T*_*m*_), which can induce significant membrane leakage and can be lethal to the cell (18, 28).

Additionally, water efflux increases the intracellular solute concentration, causing rapid membrane shrinkage and volume loss, ultimately leading to macromolecular crowding as cellular components are forced into closer proximity. Increased macromolecular crowding alters molecular diffusion rates and negatively affects proteostasis networks. Monovalent cations, such as potassium (K^+^), are the most abundant solutes present in cells, and, under increasing osmotic stress, the intracellular concentration of these ions increases rapidly (29–31). Although the influx of monovalent cations is effective at restoring the osmotic pressure gradient, this large increase in intracellular ion concentration can prevent the intra- and intermolecular electrostatic interactions that stabilize proteins’ three-dimensional structures (32–34). Additionally, the abnormal exposure of hydrophobic residues during protein unfolding results in the promiscuous sticking together of proteins (35, 36). These phenomena, which are linked to the loss of the molecular hydration layer that enables hydrogen bonding, can lead to further protein misfolding and increased protein aggregation.

Furthermore, a direct result of increased molecular crowding and protein misfolding is the disruption of important metabolic networks. As intracellular diffusion rates decrease due to crowding, enzymes are unable to reach their substrates, preventing metabolic reactions from occurring (34, 37). Additionally, many of the enzymes required for carrying out these metabolic processes are likely to misfold and aggregate as described above, preventing them from carrying out their functions (38). Membrane damage experienced during desiccation also alters the availability of important membrane-bound complexes involved in electron transport and other metabolic processes. Beyond enzyme limitation and membrane damage, water loss broadly limits metabolic chemistry. As a result, a large proportion of essential reactions cannot be completed.

Decreased intracellular volume also results in a relative increase in the local concentration of reactive oxygen species (ROS) (39). ROS are highly reactive oxygen-containing molecules, namely peroxides, superoxides, and hydroxyls, which can induce DNA damage through the oxidation of bases, damage to the sugar backbone, and formation of DNA cross-links (40). In bacteria, endogenous ROS are typically produced following disruption of the electron transport chain, as well as through Fenton reactions (34, 41, 42). For this reason, ROS generation is tied to the specific metabolic changes induced by desiccation, as outlined above. Furthermore, the rapid resumption of metabolism during the initial stages of rehydration may result in a sudden increase in ROS as oxygen consumption occurs (43, 44). The accumulation of ROS during desiccation and rehydration, and the increased likelihood that these molecules will interact with DNA due to intracellular crowding, creates a significant stress for the cell.

## KNOWN MECHANISMS OF DESICCATION TOLERANCE IN BACTERIA

**Fig 1:**
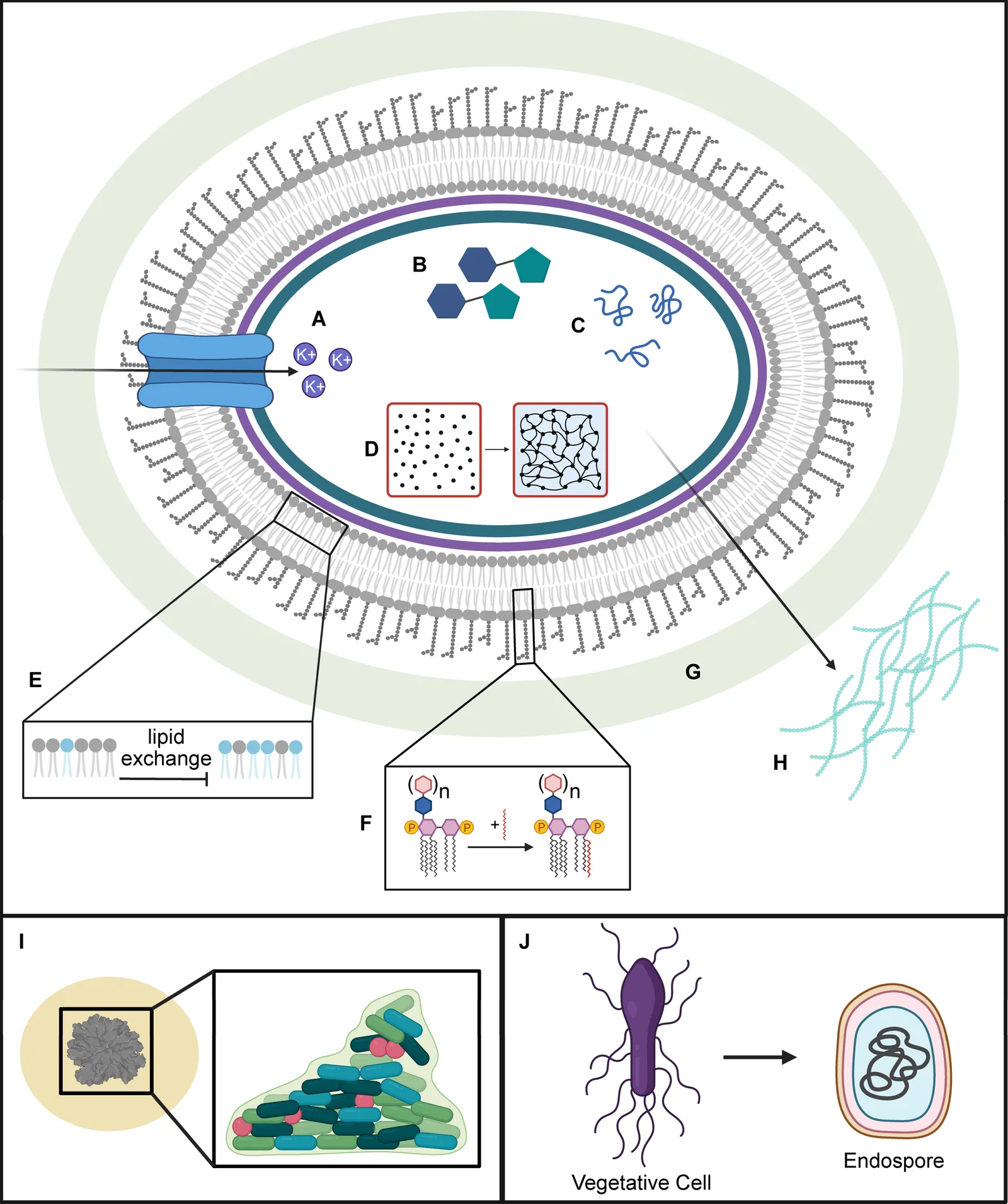
General mechanisms of desiccation tolerance in bacteria. A diverse set of mechanisms for tolerating desiccation stress have been proposed in bacteria, including (**A**) ion import, (**B**) accumulation of compatible solutes, (**C**) production of intrinsically disordered proteins, (**D**) cytosolic vitrification, (**E**) preventing the exchange of unsaturated membrane lipids for saturated lipids, (**F**) lipopolysaccharide modifications, including acylation of Lipid A, (**G**) production of capsular polysaccharides, (**H**) secretion of extracellular polysaccharides, (**I**) formation of single or multispecies biofilms on dry surfaces, and (**J**) spore formation. Created in BioRender. Bucher, M. (2026) https://BioRender.com/75q8uhu.

Given the wide range of stressors imposed by desiccation, bacteria depend on an equally diverse set of tolerance mechanisms, as outlined in Fig. 1. Although these mechanisms have evolved independently many times across the tree of life, they often result in remarkably similar strategies. These generalized mechanisms, therefore, likely represent universally effective ways of dealing with desiccation stress.

### Cytoplasmic adaptations

In the initial stages of drying, bacteria rapidly import monovalent cations, such as K^+^, to increase intracellular solute levels and partially negate the strong osmotic pressure gradient induced during desiccation (Fig. 1A) (30). In concert, cells produce glutamate to counter the charge of the K^+^ ions (45, 46). The potassium glutamate salt then serves as a signaling molecule that induces non-ionic osmoprotectants (46, 47). The production of these non-ionic osmoprotectants, or compatible solutes, is an essential step in the desiccation stress response in bacteria (Fig. 1B). As outlined above, the accumulation of ionic osmolytes, such as K^+^, can disrupt protein stability by interfering with electrostatic interactions. As such, it is important for the cell to shift from importing ionic osmolytes to the production of non-ionic osmoprotectants, which do not interfere with protein stability, and provide additional protection (29, 48). Compatible solutes include amino acids and amino acid derivatives, such as proline, carnitine, and glycine betaine, which primarily function to maintain osmotic pressure gradients (26, 49), as well as non-reducing disaccharides such as trehalose and sucrose. Non-reducing disaccharide osmoprotectants have been shown to participate in a variety of cell protective mechanisms, including maintenance of the osmotic pressure gradient, stabilization of cell membranes (50, 51), and formation of a protective vitrified cytoplasm (25, 52). These non-reducing sugars can comprise up to 50% of the dry weight of cells during desiccation (53). These sugars stabilize the cell membrane by replacing the water molecules that typically interact with the polar head groups of phospholipids (51) and promote glass formation in the cytoplasm of whole cells.

Another component of the cell’s response to drying includes the expression of intrinsically disordered proteins (IDPs) (Fig. 1C). These proteins do not maintain a stable tertiary structure and instead dynamically interconvert between numerous conformations (54, 55). A specific category of IDPs, called hydrophilins, has been specifically linked to the promotion of desiccation tolerance (7, 55–58). Hydrophilins are often enriched in charged, hydrophilic amino acids, and contain repeating regions of disorder-promoting residues (56). Although the specific mechanisms by which hydrophilins protect cells from desiccation stress are not fully understood, it has been shown that these proteins can protect enzymes from desiccation and heat-induced inactivation (7, 59), contribute to the formation of a protective vitrified state (60, 61), and form hydrogen bonds with cellular components, replacing the role of water in stabilizing proteins and membranes (55, 62). Additionally, certain hydrophilins can synergize with non-reducing disaccharides, such as trehalose, to amplify the protective effects of these mechanisms (63). These hydrophilin proteins have been discovered across all kingdoms of life, including in the bacterial species *A. baumannii*, *Mycobacterium tuberculosis*, and *Escherichia coli* (7, 64, 65). The detection of hydrophilin proteins across such diverse organisms suggests that further screening will result in the identification of additional hydrophilin proteins in other bacterial species.

Formation of a cytosolic vitrified or glassy state under extreme desiccation is an unavoidable consequence of water loss and can be either protective or non-protective to the cell (Fig. 1D) (53, 66). In the vitrified state, intracellular molecular motion is slowed, limiting the rate at which proteins unfold and membranes destabilize. In this state, cells can remain dormant until they are rehydrated (34). In desiccation-tolerant organisms, the state of the cytosol before vitrification is protective, and the accumulation of non-reducing sugars and hydrophilins, which shield membranes, proteins, and other cellular components, primes the cytosol for the formation of protective glasses. Prior to cytoplasmic vitrification, desiccation-tolerant organisms also induce the production of DNA repair and oxidative stress response proteins (67), allowing the cell to rapidly manage the stress that ensues as the vitrified state collapses under rehydration.

### Cell envelope adaptations

Bacteria can alter the lipid composition of their membranes to improve desiccation tolerance by preventing the depletion of unsaturated lipids and the associated increase in the *T*_*m*_ that occurs during drying (68–71) (Fig. 1E). By resisting increases in the *T*_*m*_, bacteria can prevent the transition to gel-phase membranes, which are prone to disruption and leakiness, exacerbating water loss and the osmotic stress induced by desiccation. Similarly, gram-negative bacteria can modify the lipopolysaccharide (LPS) layer of their outer membranes (OM) to promote desiccation tolerance (72). These modifications are typified by the addition of acyl chains to the Lipid A (Fig. 1F) (72). Acylation of the Lipid A component of LPS increases OM packing and decreases permeability (72, 73), thereby reinforcing the membrane during desiccation stress. In doing so, gram-negative organisms can decrease membrane fluidity, therefore reducing the rate at which water diffuses out of the cell during drying.

Finally, some bacterial species produce capsular polysaccharides that protect the cell against a variety of environmental stressors, including water loss. Under desiccation stress, capsule production is often upregulated, and cells with a thicker capsule are more tolerant to desiccation (Fig. 1G) (74). The hydrophilic nature of capsular polysaccharides can enhance water retention during desiccation, providing a potential mechanism for this observed benefit (25). Similarly, bacteria can produce extracellular polysaccharides (EPS), which are secreted from the cell and form a gel-like matrix (Fig. 1H) (75). The formation of this matrix limits water loss during desiccation, promoting tolerance (76, 77). These EPS molecules are core components of the bacterial biofilm (78). The formation of biofilms under dry conditions has recently been explored in numerous bacterial species (Fig. 1I). However, the importance or essentiality of dry surface biofilm formation in desiccation tolerance is not well understood despite evidence that this strategy is employed by several nosocomial pathogens, including *Enterococcus faecium*, *Klebsiella pneumoniae*, and *S. aureus* (79–81).

### Sporulation

Some gram-positive bacteria, including the pathogens *Clostridioides difficile*, *Bacillus cereus*, and *Bacillus anthracis*, are capable of sporulation, or the formation of a protective, dormant spore through asymmetric cell division, which is resistant to many external stressors, including extreme desiccation (82) (Fig. 1J). The resilience of spores to desiccation is due to the partially dehydrated nature of the spore core and the high concentration of dipicolinic acid (DPA) present in the spore core (83–85). The dehydrated nature of the core limits the further loss of water under desiccation stress. Additionally, the accumulation of DPA can mitigate the effects of water loss by binding to calcium (Ca^2+^), thereby reducing membrane permeability and restricting the mobility of macromolecules (86, 87). Moreover, high levels of acid-soluble spore proteins (SASPs) have been shown to bind and protect DNA within the spore core and are important in protecting the spore from desiccation-induced DNA damage (83, 85). These intrinsically disordered SASPs lack a defined tertiary structure until they bind DNA under desiccation-induced stress, suggesting one of many potential mechanisms by which IDPs contribute to desiccation tolerance (88).

## DESICCATION TOLERANCE IN NOSOCOMIAL PATHOGENS

Desiccation tolerance is significant for the transmission of nosocomial pathogens, which often form reservoirs on abiotic surfaces in hospital settings. Although the mechanisms of desiccation tolerance are well-characterized in desiccation-tolerant eukaryotic organisms and some extremophile bacteria, comparatively little is known regarding the mechanisms by which bacterial pathogens tolerate water loss. Nevertheless, studies of clinically relevant bacterial species have begun to identify key mechanisms that drive desiccation-tolerance phenotypes in these organisms, providing an initial framework for understanding their responses to water limitation. Here, we summarize the desiccation tolerance profiles of five important nosocomial pathogens, *A. baumannii*, *Enterococcus* spp., *C. difficile*, *P. aeruginosa*, and *S. aureus*, and discuss known protective mechanisms employed by these organisms to survive drying. It is important to note that there is substantial variation in the reported desiccation survival times of these pathogens, likely due to experimental variation in a variety of factors that can affect desiccation tolerance, including surface material, temperature, growth stage, relative humidity, and inoculum size (Table 1) (89, 90).

**TABLE 1:** Reported desiccation survival times and relevant experimental conditions for the five discussed nosocomial pathogens

Bacteria	Survival time	Conditions	Temperature and relative humidity	Source
*Acinetobacter baumannii*	3–12 days	Stainless steel, Formica, drip stand, inoculum of 10^6^ cells	20°C–22°C, 60%–70% RH	(91)
	>7 days	Towel, polyester, different inoculum sizes	25°C, 52% RH	(92)
	7–19 days	Cotton, cotton-polyester, wool, silk	21°C–24°C, 30%–49% RH	(93)
	≤20 days	Glass coverslips, inoculum of 10^7^ cells	RT, 10% or 31% RH	(94)
	21–33 days		22°C, 31% RH	(95)
	18–60 days	Glass coverslips supplemented with BSA at different humidity levels, inoculum of 10^7^ cells	20°C–24°C, 10%, 31%, or 93% RH	(96)
	28 days	Phone surface, inoculum of 10^6^–10^7^ cells	20°C–22°C, 40%–50% RH	(97)
	>28 days	Glass, stainless steel, polyvinyl chloride, and aluminum	28°C, 31%–35% RH	(98)
	>28 days	Petri dishes, inoculum of 10^9^ cells	20°C, 40% RH	(99)
	>30 days		22°C–24°C, 45% RH	(100)
	>60 days	Glass coverslips, lab coat, plastic, inoculum of 10^7^ cells	RT, 54% RH	(101)
	>60 days	Different fabrics and plastic surfaces, different inoculum sizes	22.5°C–26.2°C, 20%–49% RH	(102)
	>90 days	Cotton	22°C, 55% RH	(103)
	>90 days	Polystyrene	19°C–23°C, 25%–61% RH	(104)
	50–100 days	Isolates from different species, tested on polystyrene	22°C, 40%–60% RH	(105)
	16–96 days	Glass coverslips, inoculum of 10^7^ cells	22°C, 31% RH	(106)
	>100 days			(107)
	>3 months	Polyvinyl chloride	22°C, 50% RH	(108)
	>250 days	Polystyrene	RT, 20%–30% RH	(7)
	329 days	Screw-top glass bottle, inoculum of 10^9^ cells	22°C–27°C, 27%–45% RH	(109)
	>2 years	Powdered infant formula, inoculum of 10^8^–10^10^ cells	21°C	(110)
*Enterococcus faecium*	<7 days	Countertops, inoculum of 10^4^ cells	Not available	(111)
	VRE from patient stool: >7 days	Countertop		(112)
	VRE from culture: >58 days			
	>7 days	Upholstery, flooring, and wall materials, inoculum of 10^5^ cells		(113)
	>7 days	Towels, polyester, different inoculum sizes	25°C, 52% RH	(92)
	21 days	Cotton, inoculum of 10^8^ cells	25°C	(114)
	>28 days	Glass, stainless steel, polyvinyl chloride, and aluminum	28°C, 31%–35% RH	(98)
	>675 h	Endotracheal tubes, inoculum of 10^4^ cells	Not available	(115)
	≤51 days	Cotton, cotton-polyester, wool, silk	21°C–24°C, 30%–49% RH	(93)
	<69 days	Microtiter plate	RT	(116)
	>84 days	Polyurethane, stainless steel, different inoculum sizes	RT, 34% RH	(117)
	22 to >90 days	Cotton, terry, fabric blend, polyester, polyethylene	22.9°C–24.5°C, 30%–49% RH	(118)
	>16 weeks	Glass slides	18°C–21°C, 40%–70% RH	(119)
		Glass bottles with silica gel particles, inoculum supplemented with skim milk	22°C	(120)
	≤4 months	Polyvinyl chloride	22°C, 50% RH	(121)
	>84 weeks	Dry biofilm on stainless steel	20°C, 55% RH	(79)
	≤1,451 days	Screw-top glass bottle, inoculum of 10^10^ cells	RT	(122)
	≤2,009 days			
*Enterococcus faecalis*	>12 h	Glass	25°C, 85% RH	(123)
	>4 days	Glass, polyvinyl chloride, stainless steel, polypropylene	25°C, 0% RH	
	<5 days	Countertops, inoculum of 10^4^ cells	Not available	(111)
	>28 days	Petri dishes	20°C, 40% RH	(99)
	<28 days	Phone surface, inoculum of 10^6^–10^7^ cells	20°C–22°C, 40%–50% RH	(97)
	43–68 days	Vinyl, ceramic tile, laminate, and inox sheet	18°C–23°C, 70% RH	(124)
	11 to >90 days	Different fabrics and plastic surfaces, inoculum of 10^5^ cells	22.9°C–24.5°C, 30%–49% RH	(118)
	3 to >8 weeks	Exponential phase cultures and blood, urine, or saliva inoculated on the floor, synthetic fibers, cotton, or mattresses, inoculum of 3.0 × 10^8^ cells	Not available	(125)
	>16 weeks	Glass slides	18°C–21°C, 40%–70% RH	(119)
*Clostridioides difficile*	≤15 min	Vegetative cells, glass slides, inoculum of 10^6^ cells	37°C,	(126)
	>24 h	Spores, glass/agar, aerobic/anaerobic, inoculum of 10^6^ cells		
	>4 weeks	Environmental contamination on multiple hospital surfaces	Not available	(127)
	≤10 weeks	Environmental contamination on hospital cubicles occupied by patients with CDI.		(128)
	>12 weeks	Spores, stainless steel	21°C–27°C, 40%–63% RH	(129)
	>5 months	Hospital floors, initial inoculation with vegetative cells	Not available	(130)
*Pseudomonas aeruginosa*	<1 h	Phone surface, inoculum of 10^6^–10^7^ cells	20°C–22°C, 40%–50% RH	(97)
	<2 h	Cotton lint, inoculum of 1.5 × 10^5^ cells	21°C, 50% RH	(13)
	2 h to 10 days	Different fabrics and plastic surfaces, different inoculum sizes	22.5°C–26.2°C, 20%–49% RH	(102)
	<1 day	Formica, inoculum of 10^4^–10^5^ cells	20°C–22°C, 30%–40% RH	(131)
	>2 days	Petri dishes, inoculum of 10^8^ cells	Not available	(132)
	>3 days	Closed suction catheters, inoculum of 10^3^ cells	Not available	(133)
	1–5 days	Stainless steel, Formica, drip stand, inoculum of 10^6^ cells	20°C–22°C, 60%–70% RH	(91)
	>7 days	Petri dishes, inoculum of 10^9^ cells	20°C, 40% RH	(99)
	2–9 days	Petri dish supplemented with saline, sputum, albumin, or sucrose. Inoculum of 10^6^ cells	25°C	(134)
	<20 days	Cotton, inoculum of 10^8^ cells	25°C	(114)
	>28 days	Polystyrene	RT, 20%–30% RH	(7)
	7–31 days	Vinyl, ceramic tile, laminate, and inox sheet	18°C–23°C, 70% RH	(124)
	33 days	Cotton, cotton-polyester, wool, silk	21°C–24°C, 30%–49% RH	(93)
	3 to >6 weeks	Paper animal bedding pieces, inoculum with or without BSA supplementation	22°C, 45%–65% RH	(135)
	3 to >8 weeks	Exponential phase cultures and blood, urine, or saliva inoculated on the floor, synthetic fibers, cotton, or mattresses, inoculum of 3.0 × 10^8^ cells	Not available	(125)
*Staphylococcus aureus*	>3 days	Stainless steel, inoculum of 10^2^–10^3^ cells	22°C	(136)
	<7 days	Formica, inoculum of 10^4^–10^5^ cells	20°C–22°C, 30%–40% RH	(131)
	>7 days	Paper, inoculum of 10^9^ cells	23°C, 55% RH	(137)
	12 days	Polypropylene, inoculum of 10^4^ or 10^6^ cells	Not available	(138)
	21 days	Cotton, inoculum of 10^8^ cells	25°C	(114)
	≤22 days	Glass, polyvinyl chloride, stainless steel, or aluminum	28°C, 31%–35% RH	(98)
	63–75 days	Vinyl, ceramic tile, laminate, and inox sheet	18°C–23°C, 70% RH	(124)
	≥60–80 days	Cotton, inoculum of 10^7^ cells	22°C–25°C, 42%–54% RH	(139)
	>8 weeks	Exponential phase cultures and blood, urine, or saliva inoculated on the floor, synthetic fibers, cotton, or mattresses, inoculum of 3.0 × 10^8^ cells	Not available	(125)
	>175 days	Plastic with the addition of dust, inoculum of 10^8^ cells	22°C–27°C, 27%–45% RH	(140)
	>1,097 days	Polypropylene	25°C	(141)

### Acinetobacter baumannii

Among nosocomial pathogens, the gram-negative bacterium *Acinetobacter baumannii* is noted for its substantial desiccation tolerance, with reported survival times ranging from days to more than 2 years of drying (Table 1). However, despite numerous studies, no single accepted model exists for how *A. baumannii* responds to desiccation. In one model, authors propose a “boom-and-bust” strategy, in which most cells die, while a small subpopulation of bacteria persists in the microenvironment created by the dead cells (142, 143). Alternatively, desiccation has been reported to induce a progressive transition of cells into a “Viable but not Culturable” (VBNC) state, accompanied by a progressive cell death over time (144–146). Together, these findings suggest that *A. baumannii* may employ multiple, non-mutually exclusive strategies to survive desiccation. This interpretation is supported by evidence of strain-dependent variation in desiccation responses, with some strains remaining culturable for extended periods, while others lose culturability but retain viability over time (147).

In addition to these broad models of tolerance, multiple specific molecular mechanisms have been shown to contribute to desiccation tolerance in *A. baumannii*. A number of global regulatory proteins have been implicated in the desiccation tolerance of this pathogen, including BfmR, the response regulator of the BfmRS master two-component system (7, 104, 148). Several BfmRS-regulated processes, including capsule production and biofilm formation, have been linked to resistance to drying, and their expression correlates with increased desiccation tolerance in both clinical and environmental isolates (149–153). Additionally, BfmR has been shown to mediate the cross-protection to desiccation induced by prior exposure to other environmental stressors, including starvation and osmotic stress (104). The RNA-binding protein CsrA is also required for desiccation tolerance in *A. baumannii* (154). This global regulator has been shown to affect the expression of at least 200 proteins, including factors involved in oxidative stress resistance and biofilm production (154). Additionally, induction of the stringent response appears to play a role in protection against drying, as mutations in a *relA* ortholog in *A. baumannii* reduce the desiccation tolerance of this organism (155).

Modifications of the bacterial envelope and OM have been implicated in the desiccation tolerance of *A. baumannii*. While most bacteria induce the addition of an extra acyl chain upon stress, *A. baumannii* constitutively expresses hepta-acylated Lipid A (72). Hepta-acylated lipooligosaccharide appears to reinforce the OM and promote tolerance to stressors, including antimicrobial agents and desiccation. Supporting this conclusion, a penta-acylated mutant of *A. baumannii* exhibited a desiccation tolerance defect, surviving for less than 48 h of drying (72). Phenotypic switching to the Vir-O (Virulent-Opaque) state, characterized by opaque colonies due to increased capsule production, has also been linked to increased desiccation tolerance in *A. baumannii* (156, 157). Consistent with a role for capsule in desiccation tolerance, increased capsule production has been observed in many clinical isolates and may explain their increased tolerance to multiple stresses (101, 158–160). In fact, several studies have observed that encapsulated strains are more resistant than non-encapsulated strains to many stressors, including desiccation (74, 156, 158, 161).

Intrinsically disordered proteins and disordered regions within proteins appear to play a key role in the desiccation tolerance of *A. baumannii*. The bacterial hydrophilins DtpA and DtpB were found to contribute to desiccation tolerance in *A. baumannii,* and the random-coiled C-terminus of the RNA chaperone Hfq has also been reported to be involved in the desiccation tolerance response (7, 154, 162). Moreover, the ATP-dependent protease Lon was found to influence the expression of *dtpA* and *dtpB,* with a ∆*lon* mutant showing increased transcription of these factors, correlating with higher levels of desiccation tolerance in this strain (7). Another report confirmed the increased desiccation tolerance of a ∆*lon* mutant and demonstrated the formation of protein aggregates in this strain (163). This report also observed that inducing proteotoxic stress with a ribosome-targeting antibiotic prior to drying induced protein aggregate formation and increased desiccation tolerance in *A. baumannii*. These findings raise the intriguing possibility that an increase in aggregated protein content may be beneficial for tolerating desiccation. While the specific mechanisms by which disordered proteins protect against drying remain poorly understood, DtpA was shown to protect enzymes against inactivation by heat and desiccation (7). These findings suggest functional similarities to the reported roles of IDPs in stress tolerance in other organisms, such as tardigrades (164).

Finally, several metabolic processes have also been implicated in the response of *A. baumannii* to desiccation. Supplementation with phenylacetic acid during growth renders cells more tolerant to desiccation, presumably because it coats the OM and OM proteins in its anionic form (165). Phenylacetic acid metabolism was also recently linked to modulating the transition of *A. baumannii* into the VBNC state in response to desiccation (145). Compatible solutes have been shown to protect *A. baumannii* from desiccation through osmoregulation, as described above (166). For example, the trehalose biosynthetic pathway is involved in tolerance to both desiccation and osmotic stress (166–169). The expression of enzymes involved in glutamate and lysine synthesis is also upregulated in *A. baumannii* after rehydration from desiccation, likely to protect against osmotic and oxidative stress (143). Similarly, increased expression of glutamate transport and synthesis genes during desiccation has been previously reported in this organism (143).

### *Enterococcus* spp.

Enterococci, in contrast to their *Carnobacterium* and *Vagococcus* ancestors, have evolved to tolerate a wide range of stresses, including desiccation, enabling them to survive the transition to terrestrial life and become important human pathogens (170). Numerous studies have evaluated the desiccation tolerance of the two primary human pathogens in this genus and observed survival for more than 16 weeks in *Enterococcus faecalis* and up to 5 years in *Enterococcus faecium* (Table 1). Although *E. faecium* is generally more desiccation-tolerant than other enterococcal species, vancomycin-resistant *Enterococcus* (VRE) and outbreak strains do not exhibit increased tolerance relative to other *E. faecium* isolates (121, 122). As with other nosocomial pathogens, only a few mechanisms underlying desiccation tolerance in enterococci have been characterized. It was reported that the loss of the metalloprotease Eep in *E. faecium* decreases desiccation tolerance (116). This membrane-bound protease is known to play a role in stress responses by degrading the anti-sigma factor RsiV, thereby liberating the sigma factor SigV (171, 172). However, the mechanism by which SigV mediates desiccation tolerance remains unknown.

The accumulation of osmoprotectants, such as glycine betaine, primes *E. faecium* cells to better survive desiccation (173). Similarly, trehalose, sucrose, and maltodextrin were found to promote the survival of *E. faecium* following drying on a cotton surface (174). Another area under investigation is dry surface biofilm formation by *E. faecium* on stainless steel, a state that enables survival under desiccation for at least 84 weeks of desiccation (79). While the desiccation tolerance of planktonic cells was not measured in this study, the formation of dry surface biofilms is associated with increased stress tolerance in other organisms, in part through entry into the VBNC state (80, 175). However, while previous work has shown that *E. faecalis* cells enter the VBNC state in response to starvation, cells subjected to desiccation may not enter such a state (79, 176, 177).

### Clostridioides difficile

The desiccation tolerance of *Clostridioides difficile* has not been widely explored despite its frequent contamination of hospital environments, which play a major role in transmission networks (130, 178–180). Sporulation is believed to be the primary driver of the persistence of *C. difficile* on hospital abiotic surfaces, as vegetative cells were found to survive desiccation for only 15 min and up to 6 h on moist surfaces, whereas spores can survive months of drying (Table 1) (126, 129). These findings are further supported by the observation that germinated spores are stable under anaerobic and wet conditions, but not under aerobic or dry conditions (181). Several groups have reported that *C. difficile* spores persist on different hospital abiotic surfaces for periods ranging from weeks to several months (2, 126–128, 130, 179). Importantly, none of these reports extended their studies beyond 5 months, leaving open the possibility that spores may survive even longer periods. Additionally, no reports have evaluated the desiccation tolerance of modern *C. difficile* isolates that display increased sporulation, such as those of ribotype 27, which may be better adapted to persist on surfaces (182). These findings highlight the importance of investigating desiccation tolerance mechanisms in bacterial spores and their role in forming reservoirs on hospital abiotic surfaces in future studies.

### Pseudomonas aeruginosa

*Pseudomonas aeruginosa* has been shown to survive desiccation for at least 2 months under certain conditions (Table 1). A Tn-seq screen identified 53 genes important for the desiccation tolerance of this pathogen, of which 22 were validated (183). These hits included genes involved in maintaining proteostasis, purine and pyrimidine synthesis, several metabolic pathways, and proteins that preserve OM integrity (183). Notably, transposon insertions in *surA*, which encodes an OM protein chaperone, and in *carB*, which encodes the large subunit of carbamoyl-phosphate synthetase, caused the largest fitness defects among the genes identified. Disruption of *surA* may alter OM protein biogenesis, resulting in changes in OM permeability and integrity (183). CarB is important for achieving the physiological conditions associated with stationary-phase growth, in which cells are better prepared to tolerate desiccation (183, 184). This diverse set of genes involved in the metabolism of DNA and proteins suggests that the turnover of damaged macromolecules is an important part of the response to desiccation. EPS production during biofilm formation has also been found to contribute to the desiccation tolerance of *P. aeruginosa* (77, 185). Specifically, increased production of the polysaccharide alginate enhances survival during desiccation by improving water retention (185). Additionally, disrupting alginate synthesis alters biofilm structure, potentially affecting water dynamics (185). A metabolic network involving biosynthetic pathways for both trehalose and α-glucans has also been determined to have a role in desiccation and osmotic stress (186). The synthesis of trehalose was shown to be important not only for osmotic stress tolerance but also as an indirect driver of α-glucan synthesis, which is a process involved in desiccation tolerance. Moreover, it was demonstrated that several types of α-glucans accumulate in the periplasm of *P. aeruginosa* in response to desiccation and that disruption of their synthesis activates the AlgU-mediated envelope stress response (187).

### Staphylococcus aureus

Desiccation tolerance in *Staphylococcus aureus* has been directly linked to increased environmental transmission and intraoperative infection risk, highlighting the importance of studying this trait as a determinant of nosocomial spread (188). The desiccation tolerance of *Staphylococcus aureus* has been investigated in both clinical and foodborne strains, with reported survival times ranging from several days to 3 years (Table 1). Transposon insertion mutagenesis and RNA sequencing approaches have identified several factors that contribute to desiccation tolerance in *S. aureus*. These include the antioxidant enzymes AhpC and KatA (189), the stress-activated sigma factor SigB, the ATPase subunit ClpX, and its protease adaptor protein YjbH (141, 190). Water retention has also been shown to be a critical factor in promoting the desiccation tolerance of *S. aureus,* as this pathogen upregulates the production of capsular polysaccharides, biofilm, and staphyloxanthin in response to water loss (190–193). These factors are all regulated by SigB-dependent promoters, which may explain the essential role of this sigma factor in desiccation tolerance. Other regulatory factors affecting desiccation tolerance in *S. aureus* include the global transcriptional regulator CodY, which is required for survival during water loss, and the transcriptional regulator TcaR, which negatively regulates desiccation tolerance in this organism (194, 195). Additionally, factors affecting biofilm formation, such as the membrane peptide transporter DtpT, have also been shown to promote tolerance to water loss (196). Finally, changes in the membrane lipid composition of *S. aureus*, driven by the housekeeping cardiolipin synthase Csl2, have been shown to protect against drying (197). Taken together, these findings underscore the importance of general stress responses in the desiccation tolerance of *S. aureus*. It is also important to note that many of these factors have been identified and validated in foodborne *S. aureus* strains, which have evolved under different pressures than those of clinical strains. Further work is therefore required to identify and validate determinants of desiccation tolerance in clinically circulating *S. aureus* isolates.

## RESPONSES TO DESICCATION DRIVE OTHER PHENOTYPES IN BACTERIAL PATHOGENS

**Fig 2:**
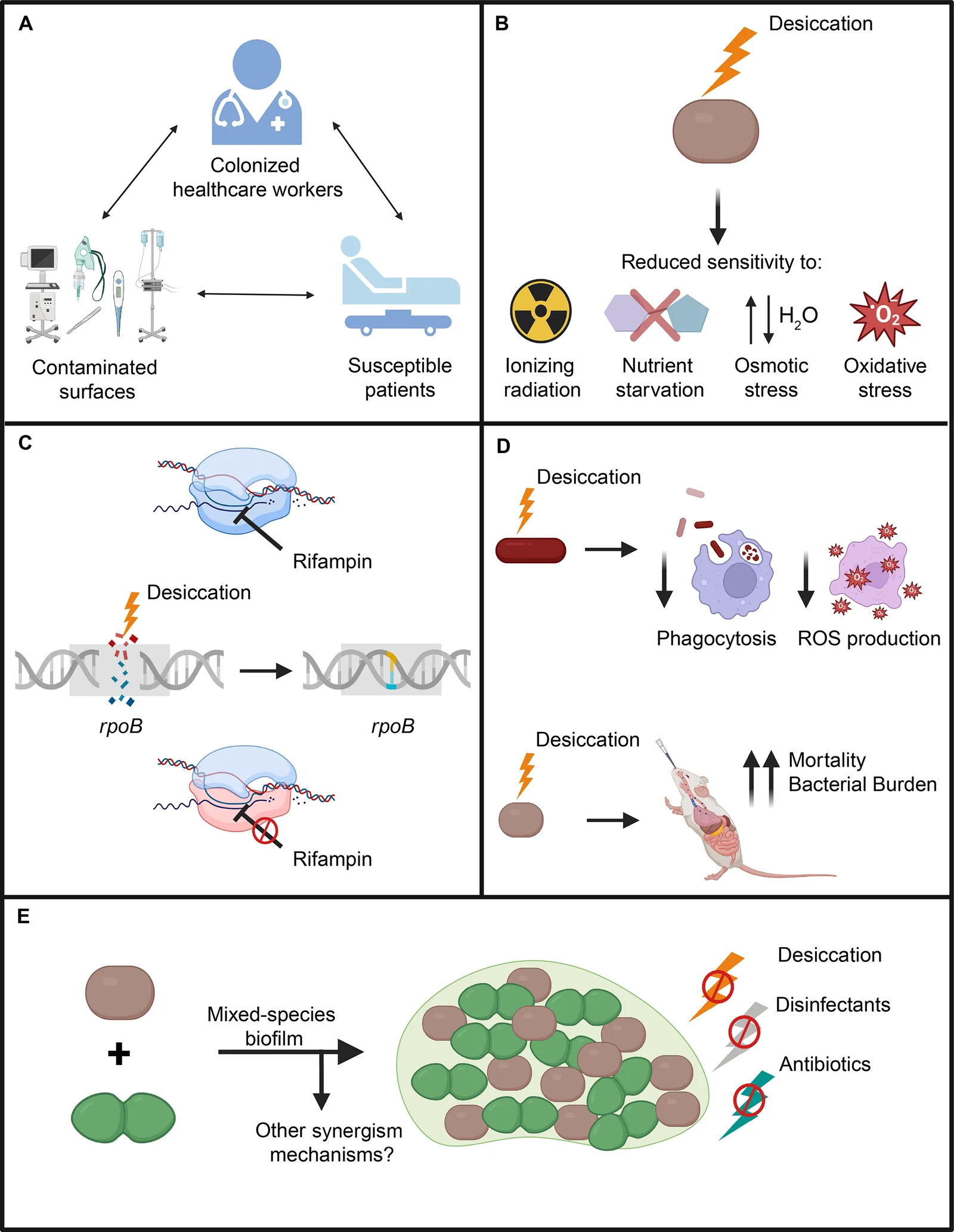
Potential benefits conferred by the response to desiccation. Emerging evidence suggests that the mechanisms conferring desiccation tolerance to bacteria may also be driving other phenotypes: (**A**) creation of transmission networks involving hospital abiotic surfaces, (**B**) induction of a cross-protective response that also allows bacteria to tolerate other stresses, (**C**) driving the evolution of antibiotic resistance through DNA damage repair, (**D**) alteration of host-pathogen interactions, and (**E**) promotion of polymicrobial synergistic interactions on surfaces. Created in BioRender. Rivera-Madera, A. (2026) https://BioRender.com/74vc2gs.

Studies of bacterial desiccation tolerance suggest that this trait contributes to pathogen persistence across a range of contexts. In healthcare settings, desiccation tolerance may promote transmission by enabling the prolonged survival of pathogens on abiotic surfaces, including medical devices and personal protective equipment, which have been implicated in pathogen spread (Fig. 2A) (2, 188, 198–200). Beyond promoting survival during drying, desiccation responses have also been shown to confer cross-protection against other stressors encountered in the hospital environment, including exposure to oxidants, antimicrobial agents, and ionizing radiation (Fig. 2B) (7, 197, 201). In addition, the formation of biofilms as a desiccation tolerance mechanism is a recurring theme among nosocomial pathogens and may indirectly promote antibiotic tolerance (151). Furthermore, exposure to osmotic stress and starvation can also increase tolerance to desiccation, highlighting the potential for crosstalk between stress responses induced by these conditions during persistence on surfaces (104, 173). Together, these observations suggest that exposure to one stress condition may prime bacteria to survive others, raising the possibility that hospital control measures may inadvertently induce broadly protective stress responses that promote surface persistence.

Another emerging idea is that mutations derived from the DNA damage incurred during desiccation induce antibiotic resistance (Fig. 2C). In *A. baumannii*, desiccation is associated with an increased frequency of *rpoB* mutations, resulting in increased levels of rifampin resistance upon rehydration (20). These mutations were found to be RecA-dependent and included variants identical to those detected in clinical isolates, demonstrating the relevance of this phenomenon in a real-world setting. Beyond its role in mutation-driven evolution, RecA mediates homologous recombination and repair and initiates the SOS response to DNA damage, making it essential for desiccation tolerance (202). Similarly, in *Mycobacterium tuberculosis,* desiccation induces oxidative stress and activates an imperfect DNA repair mechanism, leading to mutations that confer rifampin resistance (21). Taken together, these data support the hypothesis that desiccation drives the evolution of pathogens, leading to the acquisition of antibiotic resistance via DNA-damage mutagenesis. Future work should examine this phenomenon across other nosocomial pathogens and clinically relevant antibiotics to confirm that it is a conserved strategy for acquiring antibiotic resistance.

Because many nosocomial pathogens persist in a desiccated state on abiotic surfaces, understanding how desiccation influences their capacity to infect new hosts is critical. Several studies have examined the infectivity of bacterial pathogens after rehydration from desiccation, as well as modeling the direct transmission of bacteria persisting on fomite surfaces. In most of these studies, bacteria rehydrated from desiccation or deposited onto fomite reservoirs maintained infectivity in animal infection models (6, 7, 203–206). Strikingly, in some instances, exposure to desiccation prior to host infection has been associated with altered host-pathogen interactions and infection outcomes (Fig. 2D). For example, following rehydration from drying, *A. baumannii* displays increased virulence in a mouse pneumonia model (7). Similarly, desiccated *Klebsiella pneumoniae* cells elicit a more muted inflammatory response in peripheral blood mononuclear cells, despite being more readily phagocytosed by CD11b^+^ human immune cells (22). In both cases, the altered phenotypes were reversible after passaging the desiccated cells in rich media, suggesting that adaptive responses induced by desiccation may confer cross-protection within the mammalian host (7, 22).

Finally, understanding how polymicrobial interactions influence desiccation tolerance represents an important yet understudied area of research (Fig. 2E). It is common for multiple pathogens to coexist on hospital surfaces, forming a dynamic hospital microbiome that responds to and is affected by the occupants of the room (207–210). Moreover, polymicrobial biofilm formation can increase the tolerance of nosocomial pathogens to disinfectants and other environmental stressors; however, its impact on desiccation tolerance remains poorly understood (211). Evidence of synergistic mechanisms that support environmental persistence on hospital abiotic surfaces is provided by spatiotemporal associations among clonal isolates of nosocomial pathogens. For example, a spatiotemporal association between *A. baumannii* and *E. faecium* was observed on intensive care unit surfaces (207). Moreover, *P. aeruginosa* strains that were co-desiccated with hospital isolates of *Arthrobacter* spp. or *Micrococcus* spp. showed greater desiccation tolerance than *P. aeruginosa* alone (23). Finally, direct interactions between influenza A virus (IAV) and *Streptococcus pneumoniae* were shown to increase IAV stabilization under desiccation stress, highlighting the potential influence of viral-bacterial interactions on fomite-mediated disease transmission (212). Together, these findings underscore the need to investigate how polymicrobial interactions influence desiccation tolerance and persistence of nosocomial pathogens on hospital abiotic surfaces.

## CONCLUSIONS

Hospital-acquired infections are an emerging global cause of healthcare-associated morbidity and mortality and are driven in part by the environmental persistence of pathogens on abiotic surfaces. In these settings, bacteria must tolerate a wide range of stressors, including the complete absence of water. Prolonged water deprivation can induce multiple forms of cellular damage, including osmotic stress, membrane destabilization, protein misfolding, macromolecular crowding, and oxidative DNA damage. Bacteria employ a number of strategies to combat these stressors, including the accumulation of compatible solutes, remodeling of the cell envelope, the expression of intrinsically disordered proteins, and the formation of biofilms. However, our understanding of how these responses function in clinically relevant nosocomial pathogens remains incomplete.

The major nosocomial pathogens reviewed here employ overlapping yet distinct strategies for tolerating desiccation. Current evidence points to important roles for global regulatory networks, cell envelope modifications, biofilm formation, and metabolic adaptation in promoting desiccation tolerance in these pathogens. However, the relative contributions of these processes vary substantially across species, underscoring the potential need for pathogen-tailored approaches to combat bacterial contamination on environmental surfaces. Additionally, many fundamental questions regarding desiccation-tolerance mechanisms in these organisms remain unresolved, including how these pathogens sense water loss, how desiccation responses are temporally regulated and coordinated, and how entry into the VBNC state affects both environmental persistence and host transmission. Moreover, many published studies have interrogated mechanisms of desiccation tolerance in lab-domesticated bacterial strains, leaving open the possibility that responses to drying might vary in contemporary clinical isolates. Future studies should therefore evaluate the desiccation tolerance of clinically relevant strains to determine whether these isolates employ distinct strategies to tolerate desiccation.

Having a deeper understanding of the mechanisms that promote desiccation tolerance in bacteria may inform strategies to target pathogens that persist on abiotic surfaces or to inhibit their capacity to infect upon rehydration, thereby disrupting or preventing the establishment of fomite transmission networks. One potential target is cells that persist in the VBNC state, which is associated with higher tolerance to stressors, including hospital-based disinfectants (213–216). Interventions that disrupt the persistence of VBNC bacterial populations on hospital abiotic surfaces may therefore reduce the risk of transmission. For example, several reports have found that *A. baumannii* requires extended periods of time and the presence of certain ions to regain clonogenicity after rehydration (144, 177). Based on these findings, cleaning procedures could be modified to include a controlled rehydration step prior to disinfection to reverse desiccation-induced physiological changes, and thereby increase bacterial susceptibility to conventional disinfectants. Finally, insights into protective responses against desiccation may also enable their exploitation for beneficial applications, such as xeropreservation. For example, a bacterial hydrophilin protein was shown to protect enzymes from desiccation- and heat-induced inactivation, suggesting a potential use of bacterial hydrophilin proteins as stabilizing agents for protein-based therapeutics (7).

In summary, desiccation tolerance is a central determinant of the environmental persistence and hospital spread of nosocomial bacterial pathogens. Elucidating the mechanisms underlying bacterial desiccation tolerance will not only advance efforts to reduce the transmission of nosocomial infections, but could also inform the development of preservation strategies for pharmaceutical and biotechnological applications.
